# Deciphering the mechanisms of oxygen transfer into a wine bottle

**DOI:** 10.1126/sciadv.aed3023

**Published:** 2026-06-19

**Authors:** Julie Chanut, Aurélie Lagorce, Jean-Marc Simon, Igor Bezverkhyy, Jean-Pierre Bellat, Régis D. Gougeon, Thomas Karbowiak

**Affiliations:** ^1^ Université Bourgogne Europe, Institut Agro, INRAE, UMR PAM, 21000 Dijon, France.; ^2^ Laboratoire Interdisciplinaire Carnot de Bourgogne, UMR 6303 CNRS, Université Bourgogne Europe, 21000 Dijon, France.; ^3^ Institut Universitaire de la Vigne et du Vin, UMR PAM, Université Bourgogne Europe, 21000 Dijon, France.

## Abstract

This study aims to deepen the understanding of how oxygen transfers through wine bottle closures and their chemical reactivity and kinetics during storage. A miniaturized bottle system was designed to enable measurements with or without model wine. Different physical and chemical mechanisms, each with its own kinetics, were revealed through oxygen permeability measurements. Four main mechanisms were identified, each occurring over different timescales. In the first days of storage, rapid equilibrium is established between the gas and liquid phases of the model wine. During the early months, oxygen diffuses from the cork cells into the gas phase in the system. Phenolic compounds are then extracted from the cork and react with the oxygen released from the sealing system into the liquid phase, leading to a decrease in oxygen content over several months. Ultimately, long-term oxygen permeation through the closure results in a gradual, continuous increase in oxygen content within the mini-bottle system.

## Teaser

Hidden pathways governing oxygen transfer and reactions inside wine bottles are revealed across multiple timescales.

## INTRODUCTION

Wine is among the few food products capable of being preserved for consumption over extended periods, sometimes spanning several decades. Even more, the evolution of wine over time is sought after to achieve a complex aromatic profile. Since their introduction around the turn of the 17th century, cork stoppers have remained the predominant closure for most of the world’s wines. Natural cork and cork-based stoppers still account for nearly 56% of the global oenological closure market (*1*). Beyond their technical role, cork stoppers positively influence consumer perceptions of wine quality (*2*, *3*). Several studies have highlighted the positive effect of natural corks compared with screw caps and synthetic closures. Wines sealed with a cork stopper were rated substantially higher in appearance, bouquet, taste, and overall quality than wines sealed with alternative closures (*2*, *3*). Moreover, cork stoppers are often perceived as a mark of craftsmanship, associated with the winemaker’s expertise. Wines sealed with cork stoppers are frequently chosen for special occasions and as gifts, reinforcing their association with premium quality and tradition (*4*–*7*).

The influence of closure on wine aging has been the focus of extensive research, demonstrating its effect on the evolution of wine’s chemical composition over time (*8*–*16*). Oenological closures are characterized by their low oxygen permeability. The gradual, limited ingress of oxygen through the stopper can promote wine maturation, enhancing its aromas and complexity. However, excessive oxygenation can cause premature oxidation, especially in white wines (*17*), ultimately impairing the wine’s sensory qualities. The oxygen permeability of various wine closures has been extensively studied, leading to a better understanding of the mechanisms of oxygen diffusion (*18*–*23*). Some studies have investigated the intrinsic properties of the closure material (*20*, *24*) as well as the entire sealing system consisting of a cork compressed into the bottleneck (*25*, *26*). During the first few months of storage, oxygen is released from inside the bottle through the cork’s internal structure because of compression after bottling. This process is known as the initial oxygen release. Afterward, the predominant phenomenon shifts to the transfer of oxygen from the external environment into the wine via the sealing system, characterized by the oxygen transmission rate (OTR). This can be divided into two variables: a transfer through the stopper and a transfer at the interface between the cork stopper and the glass bottleneck. Additional factors, such as surface treatments or the application of overcapping wax, have also been considered, as they can significantly influence oxygen transfer through the sealing system, particularly at the glass-cork interface (*27*, *28*). A very recent study has focused on the evolution over time of the transfer properties of the sealing system over a 24-month storage period under various wine storage conditions using a miniaturized bottle system. The findings highlight that the oxygen diffusion coefficient of the stopper alone remains unchanged regardless of storage conditions (temperature and the presence of model wine) over 24 months. However, this study demonstrated that the glass-cork interface is a major pathway for oxygen entering bottled wines. At 20°C, the presence of model wine enhances oxygen transfer at the glass-cork interface, accounting for nearly 75% of the total oxygen transfer compared to the sealing system studied without model wine. At higher storage temperatures (35° and 50°C), changes in barrier properties are observed, with a significant increase in transfer at the glass-cork interface after a few months (*29*).

However, extrapolating these findings to standard wine bottles and developing a comprehensive oxygen-transfer model across the entire sealing system require further investigation, particularly regarding the stopper’s hydration state. Direct contact between the wine and the stopper may alter the mechanism of oxygen transfer, potentially affecting the cork’s sealing performance. This aspect has received limited attention in previous works. Accordingly, the present study aims to better understand the different mechanisms of oxygen transfer through the closure system and the corresponding kinetics involved during wine bottle storage. To that end, oxygen permeability through cork stoppers of varying lengths was studied. Kinetics of oxygen transfer and reactivity were followed over 18 months using microagglomerated stoppers compressed into a hermetically sealed mini-bottle system, both in the presence and absence of model wine. This original approach provides a new perspective on how wine and cork interact and their effect on oxygen transfer mechanisms during bottle storage.

## RESULTS AND DISCUSSION

### Effect of cork length on oxygen transfer in the absence of model wine

**Fig. 1.**
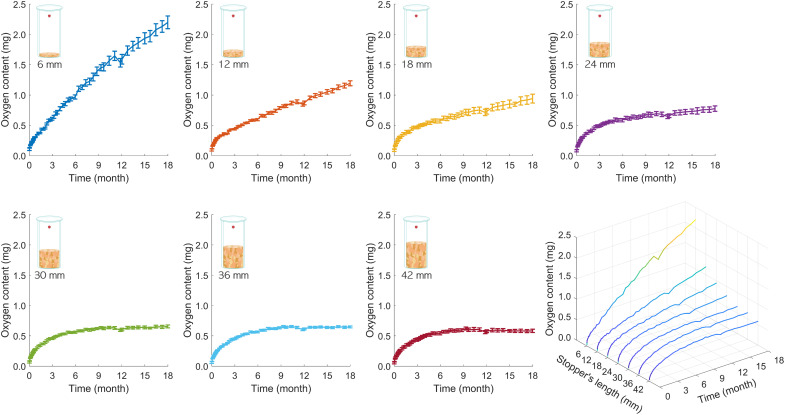
Oxygen content inside the mini-bottle system sealed with microagglomerated cork stoppers of varying lengths in the absence of model wine. The blue curve represents a 6-mm cork stopper, the orange curve represents a 12-mm cork stopper, the yellow curve represents an 18-mm cork stopper, the violet curve represents a 24-mm cork stopper, the green curve represents a 30-mm cork stopper, the cyan curve represents a 36-mm cork stopper, and the red curve represents a 42-mm cork stopper. The final graph illustrates an overview of oxygen ingress over time for all cork stopper lengths.

Oxygen ingress into the mini-bottle system in the absence of model wine was monitored over 18 months for seven different cork lengths, ranging from 6 to 42 mm. The results are summarized in Fig. 1. The oxygen transfer kinetics revealed two distinct phases, which correspond to two simultaneous mechanisms. Using data from various cork lengths helps to distinguish these mechanisms. First, a rapid increase was observed within the initial 6 months. This increase can be attributed to the release of oxygen from the cork cells to the gas phase inside the system. This phenomenon differs slightly from the so-called oxygen initial release already described in a previous study (*28*). In the present case, it is not caused by overpressure inside the cork cells resulting from compression of the stopper in the bottleneck. It is worth noting that all samples were stored for several weeks after corking before measurement. Thus, the observed oxygen release was solely driven by the difference in oxygen partial pressure between the air in the cork cells and the argon-filled interior of the system. Both were initially equilibrated at a total pressure equivalent to atmospheric pressure. The first stage of oxygen transfer was followed by a second phase, from 6 to 18 months, characterized by a slowdown in the oxygen intake (Fig. 1). In this stage, the increase in oxygen content appeared linear, with a slope that depended on the length of the stopper. The longer stopper lengths show the lowest slopes, as seen clearly for lengths between 6 and 24 mm. This second phase can be associated with the transfer of oxygen from the external environment into the system. This oxygen transfer can occur through the cork stopper and at the interface between the stopper and the glass tube (*27*, *29*).

The corresponding oxygen content, from the kinetics provided in Fig. 1, is also detailed in the Supplementary Materials (table S1) for 6-, 12-, and 18-month storage periods and for each different cork length. The highest amount of oxygen transferred after 18 months was observed for the 6-mm cork sample, with a value of 2.2 mg. For corks with lengths of 12 and 18 mm, the average oxygen quantities were around 0.8 and 0.7 mg, respectively. Between 30 and 42 mm in length, at 18 months, no significant difference was noticeable (*P* ≥ 0.05), with an average value of 0.6 mg. Therefore, beyond 30 mm in length, in the absence of model wine, the effect of the cork length on the oxygen transfer through the sealing system was below the measurement limit of the experimental procedure at 18 months (0.03% oxygen).

After the initial 6-month period, which included a mechanism of oxygen release from the cork, the OTR was determined from the pseudo–steady-state phase between 6 and 18 months. It represents oxygen transfer through both the cork stopper and glass-cork interface. The OTR values for different cork lengths are summarized in Table 1. They range from 0.01 to 1.15 mg year^−1^. The values obtained are also consistent with those previously reported for similar stoppers (*21*, *24*). The highest OTR values correspond to the lowest cork lengths. Moreover, OTR values decrease with increasing cork length. For the longest cork lengths (30 to 42 mm), the OTR values are very low, resulting in only a minimal increase in oxygen pressure within the system over the past 12 months. For such lengths, it can be assumed that either the outgassing phase is still ongoing or the transfer phase has not yet begun.

**Table 1.** Oxygen transfer parameters in a microagglomerated cork stopper in the absence of model wine. OTR and the corresponding effective oxygen diffusion coefficient values are reported for microagglomerated cork stoppers of varying lengths under dry conditions (i.e., without model wine). Values in brackets correspond to minimum and maximum values. One-way ANOVA followed by Tukey’s post hoc test was performed. Values sharing the same superscript letter are not significantly different, whereas values with different letters differ significantly (*P* < 0.05).

Cork stopper length (mm)	O_2_ transfer	
	OTR (mg year^−1^)	$D_{O_{2\;\text{total}}}$ (× 10^−11^ m^2^ s^−1^)
**6**	1.15^a^ (1.06–1.24)	1.14 (1.03–1.28)
**12**	0.56^b^ (0.52–0.58)	0.85 (0.78–0.88)
**18**	0.34^c^ (0.30–0.40)	0.75 (0.67–0.91)
**24**	0.16^d^ (0.13–0.19)	0.48 (0.40–0.55)
**30**	0.07^e^ (0.04–0.08)	0.26 (0.17–0.29)
**36**	0.05^e^ (0.04–0.06)	0.25 (0.22–0.30)
**42**	0.01^e^ (<0.01–0.02)	0.04 (<0.01–0.12)

### Influence of the wine on the oxygen transfer

#### Evolution of the oxygen content in the gas phase

**Fig. 2.**
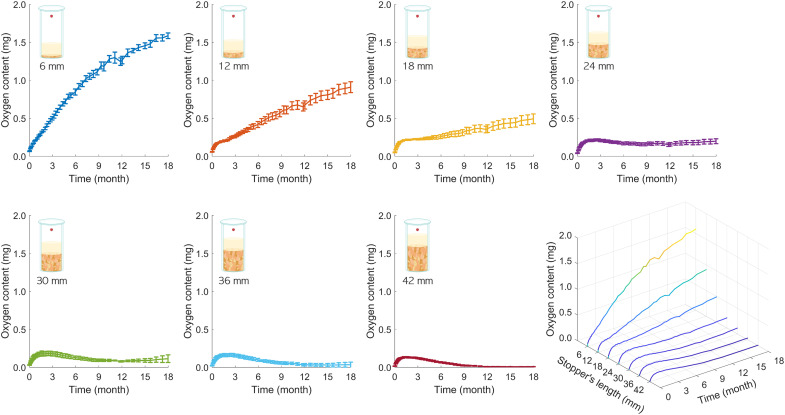
Oxygen content in the gas phase of the mini-bottle system sealed with microagglomerated stoppers of varying lengths in the presence of model wine. The blue curve represents a 6-mm cork stopper, the orange curve represents a 12-mm cork stopper, the yellow curve represents an 18-mm cork stopper, the violet curve represents a 24-mm cork stopper, the green curve represents a 30-mm cork stopper, the cyan curve represents a 36-mm cork stopper, and the red curve represents a 42-mm cork stopper. The final graph illustrates an overview of oxygen ingress over time for all cork stopper lengths.

In the presence of model wine, the observed phenomena became more complex. Different cork stopper lengths were still considered to clearly distinguish the different phenomena observed in the system. First, focusing on the evolution of oxygen pressure in the gas phase (Fig. 2), a significant increase was observed over the first 4 months. As previously noted for the mini-bottle system without model wine, this effect was more pronounced for longer cork stoppers. This can also be attributed to the release of oxygen from the cork cells into the inerted system. After this initial period, different behaviors were observed depending on the length of the stopper. For the short lengths (6 to 18 mm), a progressive increase in oxygen partial pressure was observed. This ingress became progressively less significant as the stopper length increased, as indicated by the corresponding oxygen content values shown in Fig. 2. At 12 months, the oxygen content in the gas phase for 6-mm cork was 1.27 mg, dropping to 0.67 and 0.36 mg for 12- and 18-mm corks, respectively (Supplementary Materials, table S1). After 18 months, the average value was 1.59 mg for 6-mm corks, decreasing to 0.91 and 0.46 mg for 12- and 18-mm corks, respectively. Conversely, for the longest cork lengths, a decrease in total oxygen content was observed, reaching values close to 0 mg after 12 months of storage, with average values of 0.03 mg for 36 mm and 0.01 mg for 42 mm. The 24- and 30-mm-length samples showed intermediate behavior, with a decrease in oxygen content in the gas phase between 4 and 9 months for the 24-mm samples and between 4 and 15 months for the 30-mm samples, followed by a very slow increase. After 18 months of storage, the average value was around 0.2 mg for the 24-mm cork and 0.1 mg for the 30-mm cork.

Thus, in the presence of model wine, two simultaneous phenomena predominated after 4 months of storage once the release of oxygen from the cork ended. The first mechanism involves the transfer of oxygen from the external environment into the system, increasing its oxygen content. A second mechanism involves the extraction of phenolic compounds from the cork and their reaction (*30*–*32*). These compounds react with the oxygen released from the sealing system in the liquid phase, ultimately reducing the amount of oxygen present in both the liquid and gas phases. For shorter cork lengths, the first phenomenon predominates. For longer cork stoppers, oxygen consumption due to extraction and reactivity with oxygen is more noticeable. Last, for 24 and 30 mm, a balance exists between the two phenomena. The extraction mechanism, followed by the reaction of cork phenolic compounds with oxygen, initially predominates. After several months, the oxygen transfer through the sealing system became the predominant phenomenon.

When wine is stored in contact with the stopper, some cork components can migrate into the wine after bottling. The extractable compounds, unbound or weakly bound to the cork cell wall, are mainly composed of aliphatic, triterpenic, and phenolic compounds (*33*). Among these, the extractable phenolic compounds in cork are mostly composed of gallic and ellagic acid derivatives, along with low-molecular-weight phenolic compounds (*34*, *35*). Protocatechuic acid, caffeic acid, vanillic acid, and vanillin were reported as cork phenols more susceptible to migrating into wine (*30*, *31*, *35*, *36*). These compounds may originate from the degradation of lignin and suberin. Such degradation can occur through physical or chemical processes during cork manufacturing or through microbial biodegradation (*37*). In a recent study, Gancel *et al.* (*38*) identified the phenols present in microagglomerated stopper macerates. The maceration process was conducted in a 12% (v/v) hydroalcoholic solution at 40°C for 10 days. The extracted compounds found in the microagglomerated cork extracts included ellagic acid, gallic acid, hexahydroxydiphenoyl (HHDP)-glucose isomers, and dehydrated tergallic acid-C-glucose isomers (*38*). The ultraviolet-visible (UV-Vis) absorbance spectra of model wines in contact with cork stoppers of different lengths, as well as that of a reference model wine, are presented in the Supplementary Materials (fig. S1). After 18 months of contact, the model wines exhibit significantly higher absorbance across the UV range, confirming the extraction of phenolic compounds and tannins from the cork stoppers over time. Several of these compounds exhibit characteristic absorption wavelengths (*30*, *35*, *39*). Ellagic acid shows absorption peaks near 250 and 370 nm. Its sugar derivatives, including ellagic acid-pentose, ellagic acid-deoxyhexose, and ellagic acid-hexose, also absorb around 250 and 370 nm. Gallic acid has a characteristic maximum absorbance near 270 nm. Ellagitannins, such as castalagin and vescalagin, display a maximum absorbance at around 250 nm. Other hydrolyzable tannins like HHDP-glucose and HHDP-galloyl-glucose and dehydrated tergallic acid-C-glucose isomers absorb around 270 to 280 nm and can also contribute to the peak near 380 nm.

The extracted phenolic compounds can then react indirectly with a certain amount of oxygen, catalyzed by metal cations [iron (Fe), copper (Cu), and manganese (Mn)], during nonenzymatic oxidation of wine, resulting in the production of quinones and hydrogen peroxide (*40*–*42*). The reaction between phenolic compounds and oxygen results in the consumption of oxygen in the system, accounting for the observed decline in oxygen levels over time (*43*).

It is noteworthy that the composition of the model wine used in this study does not correspond to what is usually found in the literature, although similar compositions have already been reported in several publications (*44*–*46*). The choice of malic acid over the commonly used tartaric acid was solely driven by the need for a stable acid that would not present a risk of precipitation during the experiment. We cannot exclude the fact that tartaric acid could interact with oxygen because of its tendency to oxidize in the presence of Fe and dissolved oxygen, and its role in the initiation of Fenton reactions (*47*, *48*). Residual amounts of Fe may also be extracted from cork (*49*, *50*). However, similarly to tartaric acid, malic acid is also involved in oxidation mechanisms, as it influences the catalytic activity of the Fe redox couple (*51*).

#### Evolution of the oxygen content in the liquid phase

**Fig. 3.**
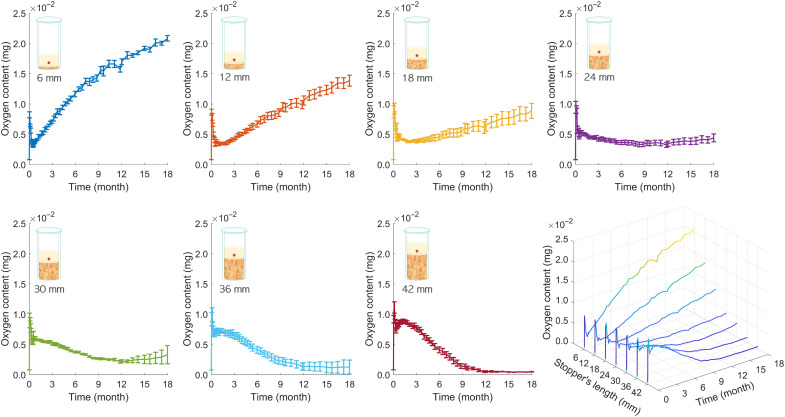
Oxygen content in the liquid phase of the mini-bottle system sealed with microagglomerated stoppers of varying lengths in the presence of model wine. The blue curve represents a 6-mm cork stopper, the orange curve represents a 12-mm cork stopper, the yellow curve represents an 18-mm cork stopper, the violet curve represents a 24-mm cork stopper, the green curve represents a 30-mm cork stopper, the cyan curve represents a 36-mm cork stopper, and the red curve represents a 42-mm cork stopper. The final panel illustrates an overview of oxygen ingress over time for all cork stopper lengths.

The evolution of the oxygen content was also followed in the model wine (Fig. 3). It displays similar behavior to the kinetics observed in the gas phase. Nevertheless, a significant difference was notable during the first 15 days. Regardless of the cork length, the oxygen content dropped rapidly in the initial days before gradually increasing again. This is likely to be due to the transfer of oxygen from the liquid phase to the gas phase in the presence of the model wine. The system tends toward equilibrium, as described by Henry’s law. Then, as previously observed, the oxygen content increased during the first 4 months because of the outgassing phenomenon by a diffusion mechanism (Fick’s law) from the cork cells to the medium in contact, driven by the chemical potential difference. This effect was more pronounced for corks of greater length. After 6 months, shorter cork stoppers (6, 12, and 18 mm) showed a gradual increase in oxygen ingress. This increase in oxygen became progressively smaller as the cork length increased.

Between 6 and 18 months, for the shortest cork lengths, the increase in oxygen content in the liquid phase can be attributed to oxygen transfer through the stopper and at the stopper-glass tube interface. At 18 months, the mean oxygen content values for samples with cork lengths of 6, 12, and 18 mm were 2.08 × 10^−2^, 1.39 × 10^−2^, and 0.89 × 10^−2^ mg, respectively (Supplementary Materials, table S1). The 24- and 30-mm samples exhibited intermediate behavior. For 24 mm, the oxygen content decreased between 4 and 9 months. The 30-mm sample showed a decrease in oxygen content between 4 and 15 months. An increase in oxygen content was then observed after 9 months for the 24-mm sample and after 15 months for the 30-mm sample. As observed in the gas phase in the presence of wine, two simultaneous phenomena predominated after 6 months of storage. Initially, the extraction of cork phenolic compounds and their subsequent reaction with oxygen were the dominant processes. However, after several months, oxygen transfer became the more prominent mechanism. Last, the oxygen content of samples with 36- and 42-mm cork stoppers declined consistently after 6 months, reaching near zero after 18 months. This decrease was attributed to the extraction of phenolic compounds from the cork, followed by their reaction with oxygen in the liquid phase. This process led to complete oxygen consumption in the system, accounting for the observed reduction in oxygen levels over time (Fig. 3).

### Oxygen exchanges in a closed system

To gain more insight into the mechanisms that occur simultaneously over time, additional measurements were conducted using a specially designed closed system with a 24-mm cork stopper. Emphasis was placed on the initial diffusion phenomenon from the cork stopper and the equilibrium between the liquid and gas phases of the model wine (closed system).

**Fig. 4.**
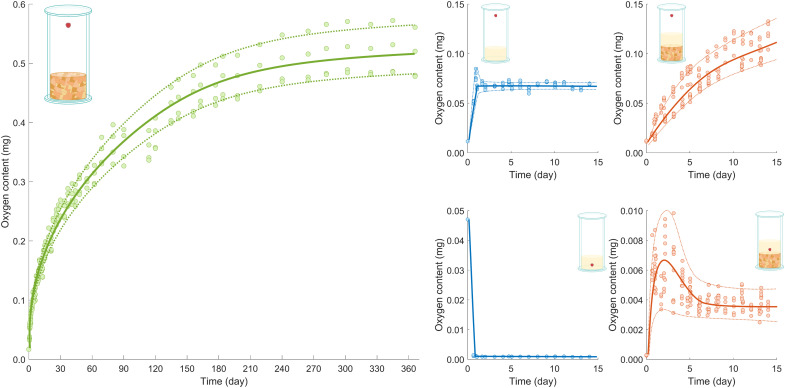
Evolution of the oxygen content over time in different closed systems. The green curve represents a closed system corked with a 24-mm-long microagglomerated stopper without model wine. The blue curves correspond to a closed system containing only nondeoxygenated model wine, with the top graph showing the gas phase and the bottom graph the liquid phase. The orange curves represent a closed system corked with a 24-mm-long microagglomerated stopper in the presence of model wine, with the top graph showing the gas phase and the bottom graph the liquid phase. In each graph, the mean value is represented by the solid line, with the minimal and maximal values indicated by the dotted lines (to guide the eye).

The kinetics of oxygen diffusion from cork were first investigated over 12 months (Fig. 4). A notable increase in oxygen release was observed during the first 3 months, reaching ~0.35 mg. This was followed by a gradual increase, with oxygen levels rising to 0.45 mg at 6 months and 0.50 mg at 9 months. The kinetic profile is similar to that of an open mini-bottle system with a 24-mm cork stopper without model wine. However, the total amount of oxygen released in the closed system was lower than in the open system, corresponding solely to the outgassing of the cork stopper. After 9 months, the oxygen release stabilized at around 0.5 mg, remaining steady until the 12th month (Fig. 4, green curve). This plateau indicates that oxygen diffusion through the cork cell walls was effectively complete by the ninth month.

The kinetics of oxygen transfer between the liquid and gas phases were then studied using a nondeoxygenated model wine solution. The equilibrium between the gas phase and the liquid phase was rapidly reached. The first measurement, taken 24 hours after sealing the system under argon, showed that equilibrium had already been reached in both phases (Fig. 4, blue curves). A similar experiment was also carried out for shorter times (data not shown). In this case, the first measurement, taken after just 30 min, showed that equilibrium had already been reached between the gas and liquid phases. As stated by Henry’s law at equilibrium, the quantity of the gas dissolved in the liquid phase is directly proportional to the pressure of the gas above the liquid. For oxygen, this can be expressed as

$${\left[O_{2}\right]}_{\text{dissolved}}=k_{H}\cdot Po_{2}$$ (1)

$$\text{With}\;k_{H}=k_{H}^{\theta }\cdot \text{exp}\left[-\frac{\Delta_{\text{sol}}H}{R}\left(\frac{1}{T^{\theta }}-\frac{1}{T}\right)\right]$$ (2)

where ${\left[O_{2}\right]}_{\text{dissolved}}$ is the concentration of oxygen in the aqueous phase (mol m^−3^), $Po_{2}$ is the partial pressure of oxygen in the gas phase (Pa), $k_{H}$ is Henry’s law constant for oxygen (mol m^−3^ Pa^−1^), $k_{H}^{\theta }$ is Henry’s law constant for oxygen at 25°C (equal to 1.3 × 10^−5^ mol m^−3^ Pa^−1^) (*52*), and $\Delta_{\text{sol}}H$ is the enthalpy of dissolution of oxygen in water at 25°C [equal to 12.06 × 10 ^3^ J mol^−1^ (*53*)]. The experimental data obtained in the liquid phase are consistent with the predictions of Henry’s law. The 30-min time frame, therefore, appears sufficient to allow complete equilibration, suggesting very rapid oxygen transfer kinetics between the gas and liquid phases for the model wine alone. This is especially noticeable considering the rather slow kinetics of the other phenomena involved in oxygen transfer in the system.

Last, the kinetics of oxygen exchange between the liquid and gas phases in the presence of a cork stopper were characterized over 15 days (Fig. 4, orange curves). In the liquid phase, an initial peak in oxygen concentration was observed 2 days after the start of the experiment. This increase was likely due to continuous diffusion of oxygen from the cork stopper into the liquid. Following this peak, the oxygen content in the liquid phase gradually declined, as oxygen transferred from the liquid phase to the gas phase, approaching equilibrium. Equilibrium between the gas and liquid phases was reached after 15 days, resulting in equilibrium values in line with predictions based on Henry’s law. Similarly, the diffusion phenomenon in the liquid phase, which lasted only a few days, can be considered negligible compared with the timescale of the other phenomena occurring during oxygen transfer.

### Modeling of the different kinetics: A balance between diffusion, reactivity, and permeation of oxygen

**Fig. 5.**
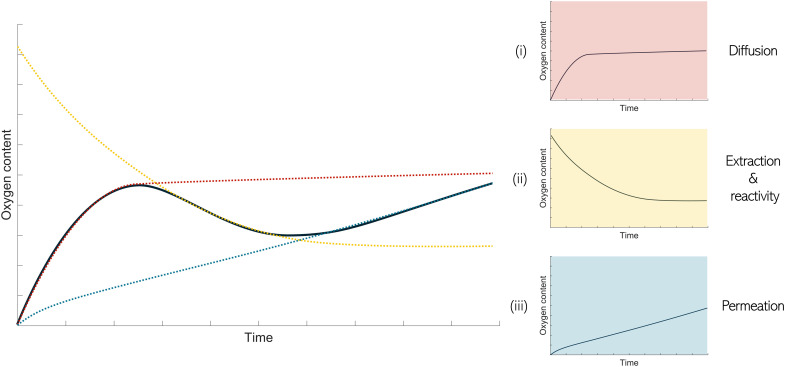
Theoretical representation of oxygen content evolution in the gas phase over time in the presence of model wine. It illustrates the three mechanisms involved and their respective kinetics. The red curve represents oxygen diffusion from the cork stopper. The yellow curve corresponds to the extraction and reactivity of cork phenolic compounds with oxygen. The blue curve depicts the permeation of oxygen from the external environment through the cork and at the interface between the cork and the glass.

The mechanisms governing oxygen transfer in the mini-bottle system, in the presence of model wine, were revealed through a series of targeted measurements. Three key phenomena were identified: (i) the diffusion of oxygen from the cork, (ii) the extraction of the phenolic compounds from the cork and their reactions with oxygen, and (iii) the permeation through the cork stopper and at the interface between the stopper and the glass. The time required to reach equilibrium between the gas and liquid phases is considered negligible compared to the other three phenomena (a few days versus several months). Figure 5 illustrates the evolution of oxygen quantity in the gas phase over time and highlights the various phenomena involved.

First, the rapid increase observed during the initial 4 months is due to the release of oxygen from the cork cells into the gas phase within the system (Fig. 5, red curve). This phenomenon can thus be attributed to a diffusion mechanism from a dense medium, in this case, the cork cell walls. The kinetics of diffusion can be described by the classical Fickian diffusion equation from a membrane of length l (Eq. 3), with a uniform initial distribution and different surface concentrations, assuming no boundary layers. In the hypothesis of a unidirectional mass transfer, this can be determined using an analytical solution to Fick’s law applied to the transient state [chapter 4, eq. 4.42 from (*54*)]

$$\frac{m_{O_{2}}-m_{O_{2\;t0}}}{m_{O_{2\infty }}-m_{O_{2\;t0}}}=1-\sum_{n=0}^{\infty }\frac{8}{{\left(2n+1\right)}^{2}\cdot \pi^{2}}\text{exp}\left[\frac{-{\left(2n+1\right)}^{2}\cdot \pi^{2}\cdot D_{O_{2}}}{4\cdot l^{2}}t\right]$$ (3)

where $m_{O_{2}}$ (g) is the quantity of the oxygen at time t, $m_{O_{2\;t0}}$ (g) is the quantity of the oxygen at the initial time, $m_{O_{2\infty }}$ (g) is the quantity of the oxygen at equilibrium, $D_{O_{2}}$(m^2^ s^−1^) is the apparent diffusion coefficient, and l is the length of the cork sample (m). Such kinetics can be described for the 24-mm sample in a closed system (Fig. 4, green curve) using the analytical solution to Fick’s law for the transient state. The oxygen diffusion coefficient can be determined in this way ($D_{O_{2}}$ value is 3.37 × 10^−11^ ± 0.14 × 10^−11^ m^2^ s^−1^). This value is of the same order of magnitude as that reported in previous studies on similar cork (*24*, *27*).

The second phenomenon observed is a decrease in the amount of oxygen in the gas phase (Fig. 5, yellow curve). This decrease is due to two concomitant processes: the extraction of phenolic compounds from the cork and their reaction with the oxygen present in the model wine. If phenolic compounds are extracted faster than they react with oxygen, the decrease in oxygen solely reflects the reaction of the phenolic compounds with oxygen present in the model wine. Conversely, if the extraction of phenolic compounds is slower than their reaction with the oxygen present in the model wine, the decrease in oxygen in the system reflects the extraction of phenolic compounds from the cork. However, the available data do not permit the determination of whether the limiting stage is the kinetics of phenolic compound extraction or their reaction with oxygen. As these two processes occur simultaneously, the overall behavior of the system is considered here. Nevertheless, it is assumed that the quantity of phenolic compounds extracted is not affected by the length of the cork. The UV-Vis absorbance spectra support this assumption, as shown in the Supplementary Materials (fig. S1), which reveals no significant differences among wines in contact with corks of different lengths. In particular, the absorbance at 280 nm (Supplementary Materials, fig. S1, red dotted lines), a parameter commonly associated with total phenolic content (*55*), remains similar for all samples. One preliminary hypothesis to consider is that the model wine had reached the saturation limit for phenolic compounds. However, the solubility of phenolic compounds, including gallic acid, in aqueous and hydroethanolic solutions has been reported in the literature to be on the order of several grams per liter (*56*–*58*). By contrast, the estimated concentrations of gallic acid equivalents in the wine samples are only a few milligrams per liter. This disparity indicates that saturation is not a limiting factor. Together, these results suggest that cork length does not influence the extraction of phenolic compounds, which likely occurs only within the first few millimeters of cork in contact with the wine.

Similar oxygen consumption trends were observed in air-saturated wines (white, rosé, and red) (*59*, *60*) as well as in air-saturated model wine enriched with phenolic compounds (*61*, *62*). They are characterized by a gradual decline in oxygen concentration over time, often approaching a threshold level or reaching zero. The decrease often follows first-order kinetics (*61*). In our study, the exponential decrease is modeled assuming a first-order kinetics equation

$$\left[O_{2}\right]={\left[O_{2}\right]}_{t0}\cdot \text{exp}\left(-k\cdot t\right)$$ (4)

$$\text{With}\;\left[O_{2}\right]=m_{O_{2}}\cdot V$$ (5)

where $\left[O_{2}\right]$ (mg liter^−1^) is the concentration of oxygen in the gas phase at time t, ${\left[O_{2}\right]}_{t0}$ is the initial concentration of oxygen in the gas phase, $m_{O_{2}}$ is the oxygen amount in the gas phase (mg), and V is the volume of the gas phase (m^3^). The determination of the kinetic constant k was based on the data from the 42-mm samples stored with model wine, as these conditions demonstrated a marked and consistent decline in the total oxygen content in the gas phase, with oxygen content approaching nearly 0 mg after 12 months of storage (Fig. 2, red curve). The kinetic fitting was specifically applied to the latter segment, beginning after 100 days of storage. Beyond this period, the influence of initial oxygen diffusion from the cork stopper is minimal, and the oxygen consumption is predominantly governed by the extraction of phenolic compounds and their reaction with oxygen. The value of k constant was estimated at 9.01 × 10^−8^ ± 1.34 × 10^−8^ s^−1^. It is also assumed that the kinetics of oxygen consumption over time are similar regardless of the stopper length. However, for shorter lengths, this rate is masked by the other two oxygen transfer phenomena.

It is worth noting that phenolic compounds found in red and white wines are present at concentrations ranging from ~200 mg liter^−1^ (expressed as gallic acid equivalents) in white wines to more than 2000 mg liter^−1^ in red wines (*63*). Numerous studies have shown that for both red and white wines, these phenolics are involved in oxygen consumption mechanisms, whether they have a positive impact (color stabilization in red wines, for instance) or a negative impact (browning in white wines, for instance). The extracted concentrations observed in this study are on the order of 1 to 10 mg liter^−1^ (gallic acid equivalents), with a significant contribution from C-glycosidic ellagitannins (*30*, *33*, *38*). Although these concentrations remain low, they are comparable to the lower concentrations in highly reactive ellagitannins extracted during barrel aging (*64*). Recent studies highlight that despite being well below the native phenolic concentrations in white wines, and negligible with respect to those in red wines, ellagitannins extracted from oak during barrel aging do contribute to the oxidative stability of dry white wines (*65*) or even to the red-to-purple color evolution of red wines (*66*). Therefore, given the fast rate of extraction observed in this study, it can be inferred that even the minor concentration of phenolic compounds extracted from cork can contribute to the consumption of the low levels of dissolved oxygen reported here.

Last, the third phenomenon is the permeation of oxygen from the outside environment into the system through the cork and the glass-cork interface (Fig. 5, blue curve). This permeation phenomenon involves oxygen sorption onto the cork surface, followed by diffusion through the material. It has already been well documented in previous studies (*20*, *24*, *27*). It can be described by the first Fick’s law of diffusion, once the steady state is established, according to Eq. 6

$$Po_{2}=\left(Po_{2\infty }-Po_{2\;t0}\right)\cdot \left[1-\text{exp}\left(-\frac{D_{O_{2\;\text{total}}}\cdot \psi \cdot S_{\text{stopper}}}{l\cdot V}\cdot t\right)\right]+Po_{2\;t0}$$ (6)

with $Po_{2}$ being the oxygen partial pressure (Pa) in the gas phase inside the mini-bottle system along time t (s), $Po_{2\;t0}$ being the oxygen partial pressure (Pa) in the gas phase inside the mini-bottle system at initial time, $Po_{2\infty }$ being the oxygen partial pressure (Pa) in the gas phase inside the mini-bottle system at equilibrium (here equal to 21,200 Pa), V being the volume of the gas phase inside the mini-bottle system (m^3^), l being the length of the stopper (m), $S_{\text{stopper}}$ being the surface of the stopper (m^2^), ψ being the separation factor or partitioning coefficient between the concentration of oxygen sorbed on the surface of the stopper and the concentration of oxygen in the gas phase, and $D_{O_{2}\;\text{total}}$ being the total effective diffusion coefficient (m^2^ s^−1^) of oxygen. ψ is obtained from the previously determined oxygen sorption isotherm on natural cork (*20*, *24*).

Effective diffusion coefficients, $D_{O_{2\;\text{total}}}$, reflecting the overall oxygen transfer within the system, were determined in mini-bottle systems, without contact with model wine, for cork lengths ranging from 6 to 24 mm, once the steady state was reached. This oxygen transfer results from the combined effects of diffusion through the stopper and along the glass/stopper interface. The results are shown in Table 1. The corresponding effective $D_{O_{2}}$ values ranged from 1.14 × 10^−11^ to 0.04 × 10^−11^ m^2^ s^−1^. A decrease in the effective diffusion coefficient is observed with increasing stopper length, which may be explained by the contribution of interfacial transfer. This interfacial effect appears to decrease with increasing length. Although slightly lower for longer lengths, the effective $D_{O_{2}}$ values obtained are consistent with those previously reported for similar stoppers (*24*, *27*).

In summary, the predominant phenomena in the mini-bottle system are governed by the storage period timescale. Initial oxygen diffusion from the cork cells into the system’s gas phase occurs during the first few months and is completed by the ninth month. At the end of the fourth month, phenolic compounds are extracted from the cork and can react with oxygen released by the closure system into the liquid phase of the model wine. Last, long-term oxygen permeation occurs simultaneously from the outside environment into the inside through the sealing system and can persist for several months to years.

The study aimed to gain a deeper understanding of the mechanisms underlying oxygen transfer through the closure system during wine storage in bottles. Specifically, it has focused on identifying the different pathways of oxygen transfer and reaction and characterizing the kinetics associated with each phenomenon, from short-term to long-term storage. To separate the mechanisms, the oxygen barrier properties of microagglomerated cork stoppers of different lengths, compressed in a mini-bottle system, were evaluated over an 18-month storage period.

Several distinct mechanisms were revealed through oxygen permeability measurements conducted both in the absence and in the presence of a model wine solution. The first mechanism observed is the rapid equilibration of oxygen between the gas and liquid phases of the model wine. This process occurs quickly, reaching equilibrium within ~15 days. Because of its rapid kinetics and short timescale, this phenomenon can be neglected when assessing long-term oxygen transfer during wine storage. Following this initial phase, oxygen diffusion from within the cork cells into the mini-bottle system is observed. This mechanism leads to a gradual increase in oxygen content and is characterized by a much slower kinetic profile that extends over 9 months. Approximately 4 months into the experiment, the oxygen concentration begins to decrease. This decrease is attributed to two concomitant processes: the extraction of phenolic compounds from the cork and their reaction with the dissolved oxygen in the wine model. As previously mentioned, these phenomena are characterized by slow kinetics, spanning over several months. In parallel with these mechanisms, oxygen permeates through the closure system from the outside environment into the inside of the system. This permeation phenomenon resulted in a gradual increase in oxygen concentration in the mini-bottle system. It persisted for several months, even years, serving as a dominant long-term oxygen transfer mechanism.

To conclude, several phenomena govern overall oxygen transfer through the closure system: initial diffusion from the cork cells, chemical reaction with phenolic compounds extracted from the cork, and long-term permeation from the external environment. Understanding the interactions and temporal overlap of these phenomena is essential for developing more advanced models of oxygen transfer in bottled wine. Future modeling work should also consider the influence of other factors, such as aging and potential changes in closure materials over time, as well as the possible dependence of the diffusion coefficient on the local hydration state of the material, on gas transfer properties.

## MATERIALS AND METHODS

### Cork stoppers

Microagglomerated cork stoppers (of the type Diam 10) were provided by Diam Bouchage (Ceret, France). They are produced by a molding process that uses cork particles and binding agents. The stoppers used in the experiments had a diameter of 24.2 mm and a length of 44 mm. They were covered with a single layer of coating consisting of a paraffin and silicone emulsion.

### Model wine

A model wine solution was prepared with the following composition: DL-malic acid (2.5 g liter^−1^), potassium sulfate (0.1 g liter^−1^), magnesium sulfate (0.025 g liter^−1^), and acetic acid (0.1 g liter^−1^). The ethanol concentration was adjusted to 12.5% (v/v), and the pH was adjusted to 3.5 with a 2 M potassium hydroxide solution (*67*). Before use, this model wine solution was inerted using nitrogen bubbling to remove dissolved oxygen. The residual oxygen concentration in the solution was below the detection sensitivity of the Pst3 sensors used to measure the oxygen content in the model wine solution (0.03% oxygen).

### Sample preparation

#### Study of oxygen transfer in an open system

**Fig. 6.**
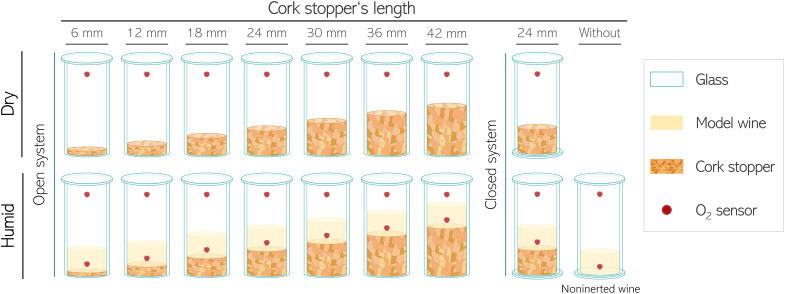
Overview of the different conditions studied and corresponding classification used for the respective samples.

Oxygen transfer was evaluated using a mini-bottle system sealed with microagglomerated cork stoppers of varying lengths. To that end, full-length corks were first inserted into glass tubes of 18.5 mm in diameter and 70 mm in length using a professional bottling machine equipped with four stainless steel jaws (GAI 4040WL, France). After corking, the compressed stoppers in glass tubes were stored for at least 4 months before the first experiments were performed. This procedure ensures that the oxygen initial release phenomenon, i.e., the release of oxygen contained in the cork cells due to compression of the cork in the bottleneck, was considered to be complete (*28*). At this stage, pressure equilibrium has been equalized on both sides of the cork cells. Cork was then partially extracted at specific distances from the glass tube using a TAX-HD+ texturometer (Swantech), yielding 6, 12, 18, 24, 30, 36, and 42 mm of the remaining compressed cork (Fig. 6). The extracted part was then trimmed. The paraffin/silicone coating layer applied to the cork stopper surface was therefore located only on the periphery of the cork and on the surface in contact with the model wine.

Chemiluminescent oxygen sensors were then fitted to the system using silicone glue Elastosil E43 (Wacker Chemical Corporation, Ann Arbor, United States) inside the glass tube. Pst3 sensors were used, which allow quantification of oxygen partial pressure ranging from 0.3 to 1000 hPa, with a resolution of ±0.1 hPa at 2 hPa. They were purchased from WQS Vinventions (Bouillargues, France). For samples without model wine, the sensor was placed 5 mm from the top of the tube and calibrated in “dry” mode, corresponding to a maximum oxygen partial pressure of 212 hPa. For samples with model wine, a first sensor was placed 5 mm from the top of the tube in the gas phase, and a second sensor was glued 5 mm from the cork, in direct contact with the liquid phase composing the model wine. These sensors were calibrated in “humid” mode, corresponding to a maximum oxygen partial pressure of 207 hPa (Fig. 6, open system).

Final sample preparation steps were performed under an inert atmosphere (Atmos bag, Sigma-Aldrich) using argon (Alphagaz 1, Air Liquide). Corked tubes without model wine were sealed by gluing a glass disc to the open end of the tube using a two-component epoxy adhesive (Araldite 2011 bicomponent, Huntsman), ensuring airtightness on this side of the system. For cork samples in contact with the model wine, 5 ml of inert model wine was added before the top part of the tube was closed with the glass disc. The corresponding residual oxygen pressure in the tube was initially around 4 hPa for all samples. For each cork length, five replicates were prepared. An external relative humidity of 50% and a temperature of 25°C were selected to study the system’s evolution under controlled conditions. This corresponds to storage under ambient conditions for temperate countries.

#### Study of oxygen exchange in a closed system

Early-stage phenomena were further investigated through additional measurements, with a particular focus on the equilibrium between the liquid and gas phases of the model wine and the initial diffusion from the cork stopper. Three specific experiments were therefore designed using a closed system. In this case, both ends of the tubes were sealed with glass discs to prevent oxygen transfer from the external environment (Fig. 6, closed system).

Kinetics of oxygen diffusion from the cork were analyzed in the first set of experiments conducted over 12 months. To that end, full-length cork stoppers were compressed in glass tubes and then partially extracted to obtain a 24-mm-long compressed cork remaining inside the tube. As in the previous case for the open system, they were left for at least 4 months after corking to allow the pressure inside the cork cells to equilibrate with atmospheric pressure. A Pst3 sensor was glued 5 mm from the edge of the tube and calibrated in “dry” mode. Last, both ends of the tube were sealed with glass discs under an argon inert atmosphere. Residual oxygen inside the tube was around 4 hPa. This experiment was conducted without a model wine, focusing solely on the kinetics of oxygen release from the cork.

Kinetics of oxygen transfer between the liquid and gas phases in the presence of model wine were investigated in the second set of experiments, while the third set focused on the role of the cork in this transfer kinetics. This was performed under two conditions: first, without the cork stopper and, second, with the cork stopper inserted in the glass tube. For samples without stoppers, 5 ml of a nondeoxygenated model wine solution was added to the glass tube, which was sealed at one end with a glass disk. Before this step, two Pst3 sensors were glued inside the tube. The first one was placed 5 mm from the bottom of the tube, in direct contact with the model wine, and the second one was placed at the other end of the tube in the gas phase. The samples were then hermetically sealed under argon with a second glass disk. Oxygen concentrations in the liquid and gas phases were monitored over 2 weeks. The same experiment was also carried out with a 24-mm cork compressed into the glass tube. In this case, the cork stopper was inserted into the tube, and 5 ml of an inert model wine solution was added. The tubes were then sealed with a glass disk. The experiments were also performed over 2 weeks, allowing for a direct comparison between the conditions with and without the cork stopper.

### Oxygen permeation

#### Measurement of oxygen transfer through corked systems

The oxygen partial pressure inside the mini-bottle systems was monitored over 18 months using an oxygen analyzer (Nomasense O2 P6000, WQS, Vinventions). The chemiluminescence method for measuring the oxygen transfer through cork stoppers has already been described by Diéval *et al.* (*23*) and Pons-Mercadé *et al.* (*68*). Nondestructive measurements were performed by positioning an optical fiber in front of the Pst3 sensor and emitting excitation light through the glass. The luminophore is excited with a sinusoidal intensity–modulated monochromatic light delivered by the optical fiber, and its decay time causes a time delay in the light signal emitted by the luminophore. This decay time decreases in the presence of oxygen and is correlated to the oxygen content (*69*). All data were collected at oxygen partial pressure (hPa) and converted to oxygen quantity (mg) for the different tested conditions.

For samples without model wine, the oxygen content was calculated as follow

$$m_{O_{2}}=M_{O_{2}}\cdot \frac{Po_{2}\cdot V}{R\cdot T}$$ (7)

with $M_{O_{2}}$ being the molar mass of oxygen (31.988 g mol^−1^), $Po_{2}$ being the partial pressure of oxygen inside the system (Pa), V being the volume inside the system (m^3^), R being the ideal gas constant (8.314 J mol^−1^ K^−1^), and T being the temperature (K). Similarly, for measurements in the gas phase in the presence of model wine, the data were obtained using Eq. 7, with V being the volume of the gas phase (m^3^).

In the case of measurements in the liquid phase, the data were converted into oxygen quantity from the dissolved oxygen concentration as follows

$${\left[O_{2}\right]}_{\text{dissolved}}=\frac{Po_{2}}{Po_{2\;\text{calibration}}}\cdot {\left[O_{2}\right]}_{\text{max},\;25{}^{\circ}C}$$ (8)

$$m_{O_{2}}={\left[O_{2}\right]}_{\text{dissolved}}\cdot V_{\text{liquid}}$$ (9)

with ${\left[O_{2}\right]}_{\text{dissolved}}$ being the concentration of oxygen dissolved in the liquid phase (mg liter^−1^), $Po_{2}$ being the equivalent partial pressure of oxygen in the liquid phase as provided by the method used (Pa), $Po_{2\;\text{calibration}}$ being the partial pressure of oxygen measured during the calibration under atmospheric pressure (and equal to 207 hPa), ${\left[O_{2}\right]}_{\text{max},\;25{}^{\circ}C}$ being the maximal concentration of oxygen dissolved in the liquid phase at 25°C (equal to 8.238 mg liter^−1^), and $V_{\text{liquid}}$ being the volume of the liquid phase (m^3^).

The measurements were carried out over 18 months to track the oxygen content entering the system as a function of time under the various conditions studied. Different sampling intervals were selected. During the first month, measurements were taken daily to capture rapid changes and early-stage dynamics. In the following 2 months, data were collected weekly to track intermediate kinetics while reducing sampling frequency. Thereafter, measurements were taken every 2 weeks for up to 9 months and, lastly, every 3 weeks to observe long-term kinetics.

#### Calculation of an OTR

Considering the gas as ideal, the surface molar flow of oxygen passing through the system, also known as the OTR and expressed in mol m^−2^ s^−1^, was calculated using the following equation, from the pseudo–steady state of the oxygen transfer kinetics

$$\text{OTR}=-\frac{1}{S}\cdot \frac{\mathrm{dn}}{\mathrm{dt}}=-\frac{V}{S\cdot R\cdot T}\cdot \frac{\mathrm{dP}o_{2}}{\mathrm{dt}}$$ (10)

where V is the volume inside the system (m^3^), S is the area of the closure system exposed to oxygen transfer (m^2^), $Po_{2}$ is the partial pressure of oxygen inside the system (Pa), R is the ideal gas constant (8.314 J mol^−1^ K^−1^), and T is the temperature (K). For practical convenience, the respective OTR was then converted into mg year^−1^ by multiplying the OTR by the molar mass of oxygen, the surface area of the compressed cork stopper, and the number of seconds in a year.

### Analysis of model wine

After 18 months of storage, the mini-bottle systems were opened by removing the glass disk sealing the end of each glass tube. The model wine in contact with the cork was collected and sealed in a glass flask under inert conditions. UV-Vis absorption spectra of the wine samples were recorded using a Genesys 50 spectrophotometer (Thermo Fisher Scientific) (*70*). Measurements were performed in UV-Vis polystyrene cuvettes (Fisherbrand), with absorbance recorded from 230 to 650 nm at 1-nm intervals, following International Organisation of Vine and Wine guidelines for the analysis of phenolic compounds in wine (*55*). The samples were diluted to 1/5 with distilled water for measurements. A model wine sample that had not been in contact with cork was used as a reference.

### Statistical analysis

Statistical analyses were carried out on oxygen content values for the different cork lengths at 6, 12, and 18 months. A one-way analysis of variance (ANOVA) with a Tukey post hoc test was performed (*P* < 0.05). The conditions for applying a one-way ANOVA were assessed, i.e., normality of residuals, homogeneity of residual variances, and independence of measurements. Statistical tests were realized using MATLAB software (MathWorks, R2019b).
