# Supplementary material for: Deciphering the mechanisms of oxygen transfer into a wine bottle
Source: Sci Adv. 2026 Jun 19;12(25):eaed3023. doi: 10.1126/sciadv.aed3023 (PMC13281788; doi:10.1126/sciadv.aed3023)
Supplement: Supplementary file 1 — Fig. S1 Table S1 [file sciadv.aed3023_sm.pdf]

Science Advances
AAAS

Supplementary Materials for

Deciphering the mechanisms of oxygen transfer into a wine bottle

Julie Chanut *et al.*

Corresponding author: Thomas Karbowiak, thomas.karbowiak@institut-agro.fr

Sci. Adv. **12**, eaed3023 (2026)
DOI: 10.1126/sciadv.aed3023

This PDF file includes:

Fig. S1
Table S1

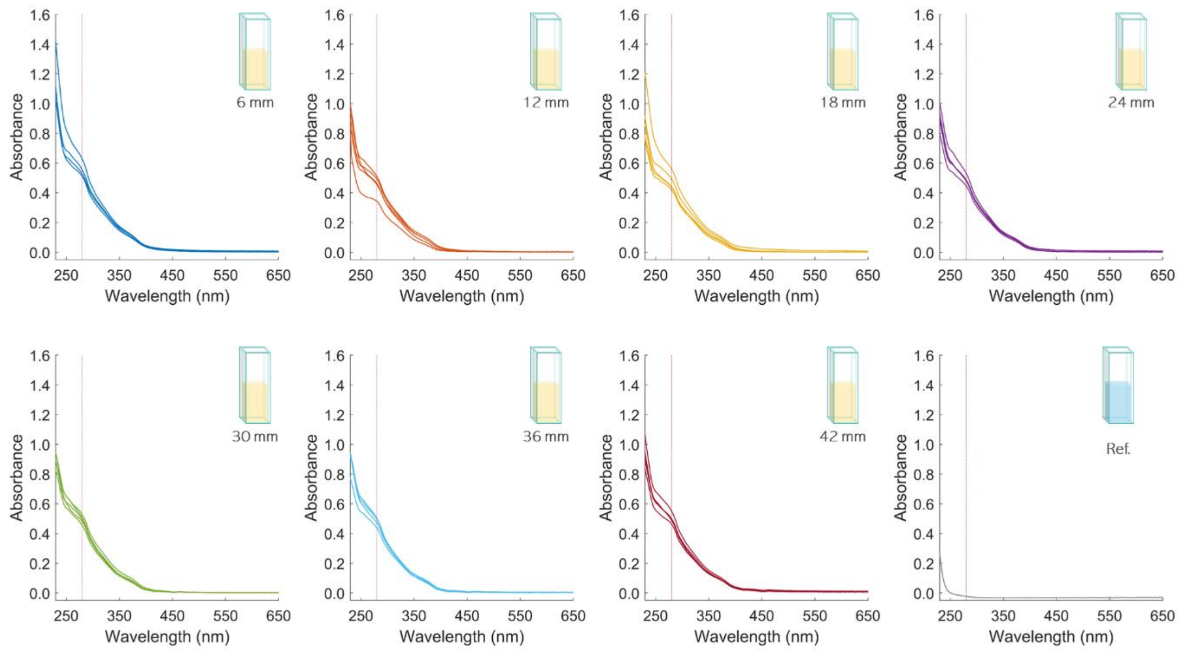

Fig. S1. UV-visible absorbance spectra of model wines in contact with cork stoppers of different lengths. The blue curve represents a 6 mm cork stopper, the orange curve 12 mm, the yellow curve 18 mm, the violet curve 24 mm, the green curve 30 mm, the cyan curve 36 mm, and the red curve 42 mm. The grey curve corresponds to the reference model wine without contact with a cork stopper. The red dotted line on each graph indicates the absorbance at 280 nm, which is used to determine the total polyphenols in wine.

Table S1. Oxygen content evolution in closed glass tubes with microagglomerated cork stoppers over 6, 12, and 18 months under different conditions. Oxygen content was measured at 6, 12, and 18 months for different lengths of microagglomerated cork stoppers compressed in a closed glass tube under three conditions: without model wine, with model wine in the gas phase, and with model wine in the liquid phase. The values in parentheses correspond to the minimum and maximum values. A one-way ANOVA was conducted on the mean oxygen quantities for each storage period, and significant differences ($P < 0.05$) are indicated by different letters.

Without model wine	Oxygen Content (mg)		
	6 months	12 months	18 months
6 mm	0.97 [a] (0.92 – 1.00)	1.59 [a] (0.82 – 1.71)	2.20 [a] (2.05 – 2.34)
12 mm	0.59 [b] (0.57 – 0.62)	0.87 [b] (0.82 – 0.90)	1.19 [b] (1.13 – 1.23)
18 mm	0.57 [b] (0.55 – 0.62)	0.73 [c] (0.70 – 0.80)	0.94 [c] (0.89 – 1.07)
24 mm	0.59 [b] (0.54 – 0.61)	0.66 [c, d] (0.96--0.68)	0.78 [d] (0.70 – 0.82)
30 mm	0.56 [b] (0.54 – 0.58)	0.60 [d] (0.59 – 0.63)	0.66 [e] (0.63 – 0.69)
36 mm	0.56 [b] (0.54 – 0.58)	0.61 [d] (0.58 – 0.63)	0.65 [e] (0.63 – 0.66)
42 mm	0.54 [b] (0.51 – 0.57)	0.58 [d] (0.55 – 0.60)	0.58 [e] (0.56 – 0.62)

With model wine	Oxygen Content in the gas phase (mg)		
	6 months	12 months	18 months
6 mm	0.85 [a] (0.79 – 0.88)	1.27 [a] (0.61 – 1.32)	1.59 [a] (1.55 – 1.62)
12 mm	0.42 [b] (0.38 – 0.46)	0.67 [b] (0.61 – 0.73)	0.91 [b] (0.83 – 0.99)
18 mm	0.26 [c] (0.23 – 0.30)	0.36 [c] (0.33 – 0.45)	0.49 [c] (0.45 – 0.61)
24 mm	0.17 [d] (0.15 – 0.19)	0.16 [d] (0.13 – 0.19)	0.20 [d] (0.16 – 0.24)
30 mm	0.13 [d, e] (0.09 – 0.14)	0.08 [d, e] (0.07 – 0.09)	0.11 [d, e] (0.07 – 0.17)
36 mm	0.10 [e] (0.08 – 0.13)	0.03 [e] (0.02 – 0.05)	0.03 [e] (0.01 – 0.08)
42 mm	0.07 [e] (0.05– 0.07)	0.01 [e] (0.01– 0.01)	0.01 [e] (0.01– 0.01)

With model wine	Oxygen Content in the liquid phase (x 10^{-2} mg)		
	6 months	12 months	18 months
6 mm	1.16 [a] (1.10 – 1.20)	1.70 [a] (0.97 – 1.75)	2.08 [a] (2.03 – 2.12)
12 mm	0.67 [b] (0.61 – 0.72)	1.06 [b] (0.93 – 1.14)	1.39 [b] (1.28 – 1.48)
18 mm	0.46 [c] (0.41 – 0.54)	0.65 [c] (0.59 – 0.82)	0.86 [c] (0.80 – 1.10)

24 mm	0.36 [d]<br>(0.33- 0.41)	0.35 [d]<br>(0.29 – 0.40)	0.44 [d]<br>(0.35 – 0.51)
30 mm	0.38 [c, d]<br>(0.35 – 0.39)	0.22 [d, e]<br>(0.20- 0.25)	0.33 [d, e]<br>(0.20- 0.49)
36 mm	0.41 [c, d]<br>(0.36 – 0.51)	0.14 [e]<br>(0.08 – 0.20)	0.13 [e]<br>(0.05 – 0.29)
42 mm	0.41 [c, d]<br>(0.33 – 0.45)	0.05 [e]<br>(0.04 – 0.06)	0.05 [e]<br>(0.04 – 0.05)
